# No Guts About It: Captivity, But Not Neophobia Phenotype, Influences the Cloacal Microbiome of House Sparrows (*Passer domesticus*)

**DOI:** 10.1093/iob/obac010

**Published:** 2022-03-11

**Authors:** T R Kelly, A E Vinson, G M King, C R Lattin

**Affiliations:** Department of Biological Sciences, Louisiana State University, Baton Rouge, LA 70803, USA; Department of Biological Sciences, Louisiana State University, Baton Rouge, LA 70803, USA; Department of Biological Sciences, Louisiana State University, Baton Rouge, LA 70803, USA

## Abstract

Behavioral traits such as anxiety and depression have been linked to diversity of the gut microbiome in humans, domesticated animals, and lab-bred model species, but the extent to which this link exists in wild animals, and thus its ecological relevance, is poorly understood. We examined the relationship between a behavioral trait (neophobia) and the cloacal microbiome in wild house sparrows (*Passer domesticus,n* = 22) to determine whether gut microbial diversity is related to personality in a wild animal. We swabbed the cloaca immediately upon capture, assessed neophobia phenotypes in the lab, and then swabbed the cloaca again after several weeks in captivity to additionally test whether the microbiome of different personality types is affected disparately by captivity, and characterized gut microbiomes using 16S rRNA gene amplicon sequencing. We did not detect differences in cloacal alpha or beta microbial diversity between neophobic and non-neophobic house sparrows, and diversity for both phenotypes was negatively impacted by captivity. Although our results suggest that the adult cloacal microbiome and neophobia are not strongly linked in wild sparrows, we did detect specific OTUs that appeared more frequently and at higher abundances in neophobic sparrows, suggesting that links between the gut microbiome and behavior may occur at the level of specific taxa. Further investigations of personality and the gut microbiome are needed in more wild species to reveal how the microbiome-gut-brain axis and behavior interact in an ecological context.

## Introduction

The gut microbiome, the collection of microorganisms living in the digestive tract of animals, has complex and bidirectional interactions with host behavior. For example, host behaviors such as dietary choices and social interactions can affect the composition of microbes in the gut (Faith et al. 2011, Godoy-Vitorino et al. 2012, David et al. 2014, Tung et al. 2015), while experimental alteration of microbial communities can in turn affect host cognitive performance and behavior (Heijtz et al. 2011, Desbonnet et al. 2014, Savignac et al. 2015, Fröhlich et al. 2016). The neural, immune, and endocrine interactions between an organism's brain and its gut microbiota is termed the “microbiome-gut-brain axis” (Clarke et al. 2013, de Palma et al. 2014, Borrelli et al. 2016, Slevin et al. 2020).

Individual personality is one of the factors that can interact with the microbiome-gut-brain axis (Davidson et al. 2018, Johnson 2020). Different individuals within a species often show repeatable variation in behavior, which has been called a personality, temperament, behavioral syndrome, or coping style (Koolhaas et al. 1999, Sih et al. 2004, Réale et al. 2007). While variation in behavioral traits has been linked to the gut microbiome in humans, domesticated animals, and lab-bred model species (Neufeld et al. 2011a, Borrelli et al. 2016, Kirchoff et al. 2019, Lee et al. 2020, Ren et al. 2020, Slevin et al. 2020, Kelsey et al. 2021), the extent to which these links exist in wild animals, and thus their ecological relevance, is poorly understood (Davidson et al. 2020a).

Neophobia describes an individual's reluctance to interact with something new or unfamiliar. This personality trait has been associated with survival (Cavigelli and McClintock 2003, Dingemanse et al. 2004, Hall et al. 2015) and several studies have shown that it may be particularly important in determining why some individuals, populations, and species are able to persist in human-altered landscapes while others are not (Candler and Bernal 2015, Greggor et al. 2016, Cohen et al. 2020). The extent to which individuals interact with novel objects and environments, and especially the extent to which they eat novel foods, may determine whether they are exposed to particular microbes (Muegge et al. 2011, David et al. 2014, Price et al. 2017, Ellison et al. 2019). Microbes in food are directly ingested, providing one potential opportunity to colonize the gut (Maul et al. 2005, Hird et al. 2014). Additionally, many animals spend large amounts of time grooming themselves and others using their mouths, which may also create opportunities for cutaneous microbes acquired from the environment to colonize the gut (Tung et al. 2015). Differential exposure could lead to differences in the diversity and composition of gut microbiomes between neophobic and non-neophobic individuals, with important consequences for host function, nutrition, and immunity (Kau et al. 2011, Cho and Blaser 2012, Foster and McVey Neufeld 2013, Rolhion and Chassaing 2016).

Our current understanding of the relationship between neophobia and gut microbial diversity in vertebrates is mostly limited to laboratory-bred mice that are diagnosed for anxiety and depression using novel object or novel environment tests. In these mice, low microbial diversity is frequently associated with reduced interactions with a novel object (Li et al. 2009, Gareau et al. 2011, Möhle et al. 2016). However, the extreme differences in microbial diversity in these animals may not be ecologically relevant (e.g., the use of germ-free mice) (Mayer et al. 2014). Therefore, whether such a relationship between neophobia and the gut microbiome exists in wild animals is not known.

In this study, we examined whether neophobia was associated with microbial diversity and community composition of the gut microbiome in wild-caught house sparrows (*P.domesticus*). House sparrows are ideal for this kind of study because they show wide and repeatable individual variation in neophobia (Bókony et al. 2012, Ensminger and Westneat 2012, Kelly et al. 2020). Previous work has also shown that non-neophobic house sparrows consume novel foods more readily than their neophobic counterparts (Martin and Fitzgerald 2005), which represents a possible mechanism by which gut microbiome differences might develop between neophobic and non-neophobic individuals.

We predicted that non-neophobic sparrows would have higher cloacal microbiome alpha diversity than neophobic sparrows, and that bacterial communities (beta diversity) would differ between the two phenotypes. We caught sparrows (*n* = 22), obtained cloacal swabs at capture, and assessed neophobia phenotypes over several weeks in the lab using novel objects and foods in a parallel study (behavior data reported in Kimball et al., in press), after which cloacal swabs were obtained again. This study design provided an opportunity to examine the effects of captivity on the microbiome, and possible interactions between captivity and personality type. We also predicted that captivity would reduce alpha diversity and change beta diversity of the cloacal microbiome in all sparrows, such that any initial microbiome differences between neophobic and non-neophobic sparrows would disappear after eight weeks of captive housing. Because captivity standardizes diet and housing conditions, studies comparing gut microbiomes in wild and captive animals generally find lower alpha diversity (i.e., richness and evenness of bacterial species) in captive samples (Xenoulis et al. 2010, Wienemann et al. 2011, Ushida et al. 2016, McKenzie et al. 2017) as well as differences in beta diversity (Ushida et al. 2016, McKenzie et al. 2017, Salgado-Flores et al. 2019, Oliveira et al. 2020). However, whether shifts in microbial diversity in captivity might differ between behavioral phenotypes is unknown. To our knowledge, this is the first study to evaluate how an animal's personality interacts with captivity to impact the gut microbiome.

## Methods

### Animal capture and husbandry

We captured adult house sparrows (*n* = 15 males and 7 females) using mist nets at bird feeders in East Baton Rouge Parish between 28 June and 16 July 2019. Sparrows were aged as adults and sexed by plumage. After extracting sparrows from mist nets, we used sterile techniques to immediately swab each sparrow's cloaca, as the cloacal community generally reflects avian urogenital and gastrointestinal microbial communities (Grond et al. 2018). Briefly, we inserted sterile swabs (#924,992, Puritan, Guilford, ME, USA) ∼5 mm into the cloaca and rotated twice (Escallón et al. 2017). We stored swabs in sterile 1.6 mL microcentrifuge tubes on dry ice until transfer to a −80°C freezer at Louisiana State University. In the lab, we housed house sparrows individually in cages in a room with a 12-h light and 12-h dark cycle and provided sparrows with *ad libitum* food (mixed seed, Mazuri small songbird diet that includes probiotics, and grit) and water, as well as a variety of perches and small dishes of sand for dust bathing. Sparrows had to be individually housed for this study because pair housing can affect neophobia (Kelly et al. 2020). Sparrows were given at least four weeks to acclimate to the captive environment before behavioral trials began. The cloaca was swabbed a second time upon completion of behavioral trials (at least eight weeks in captivity) and sparrows maintained in the lab as part of another study examining neurobiological differences between neophobic and non-neophobic individuals. Behavior results showing clear differences between neophobic and non-neophobic house sparrows in time to feed in the presence of novel objects, time to eat novel foods, and ability to habituate to the presence of a novel object are reported elsewhere (Kimball et al., in press). Briefly, we ranked sparrows in order of least neophobic (shortest average time to feed in the presence of four novel objects) to most neophobic (longest average time to feed in the presence of four novel objects). A finite mixture model analysis (Bordes and Chauveau 2016) and Weibull distribution of our data determined that an appropriate threshold to classify neophobia groups was 0.5, so the 50% slowest to eat in the presence of novel objects were deemed “neophobic” and the 50% fastest were deemed “non-neophobic.” The average time to eat in the presence of a novel object for each group (± standard deviation) was 2947 s ± 578 s for neophobic sparrows and 817 s ± 549 s for non-neophobic sparrows. Research on other birds has generally found that neophobia is consistent between laboratory and wild environments (Herborn et al. 2010, Jablonszky et al. 2020). Animals were collected under Louisiana state permit LNHP-18–098, and all experimental procedures approved by the Louisiana State University Institutional Animal Care and Use Committee. We used approved methods for sparrow capture, transport, handling, and husbandry as specified in the Ornithological Council's Guidelines to the Use of Wild Birds in Research (Fair et al. 2010).

### Microbiome sample preparation and sequencing

We tested for microbiome differences between the neophobic and non-neophobic sparrows (*n* = 11 of each pre- and post-captivity, *n* = 44 total samples). We used QIAamp® PowerFecal® Pro DNA Kits (Qiagen #51,804) to extract total microbial DNA from cloacal swabs as well as from four control samples (handled in the same manner but not inserted into a cloaca) following manufacturer's instructions. Next, we quantified DNA concentrations using a NanoDrop 2000 (Thermo Fisher Scientific). To verify the presence of DNA in low yield (<10 ng/μl) samples, we amplified the 16S region of the rRNA gene in bacterial DNA using Bakt_341F and Bakt_805R primers (CCTACGGGNGGCWGCAG and GACTACHVGGGTATCTAATCC, respectively) (Herlemann et al. 2011). We performed PCR in a C1000 Touch Thermocycler (Bio-Rad) using 10 μl volumes, which included: 5 µl DreamTaq Green PCR Master Mix (Thermo Fisher Scientific K1081), 2 µl template DNA, 2.88 µl D/RNAse free water, and 0.06 µl each of Bakt_341F and Bakt_805R primers. Thermocycling conditions included an initial denaturing step of 94°C for 3 min, followed by 30 cycles of: 94°C for 30 s, 50°C for 30 s, and 72°C for 45 s, and a final extension of 72°C for 10 min. We visualized the amplicons on a 1% agarose gel stained with 2.5 μl RedSafe™ Nucleic Acid Staining Solution (Bulldog Bio) under UV light after electrophoresis for 40 min at 100V/2A. All low-yield samples (31 of 44) showed clear amplification of the 16S rRNA gene. Genomic DNA was submitted to Michigan State University's Research Technology Support Facility, then subjected to amplification with primers modified with Illumina adapters. The library preparation for the 16S-V4 region employs a one-step PCR method using the primer pair 515F/806R: 16S V4 forward (515f): GTGCCAGCMGCCGCGGTAA, 16S V4 reverse (806r): GGACTACHVGGGTWTCTAAT (Kozich et al. 2013). Sequencing was conducted on a Miseq platform using a v2 reagent cartridge for a 2 × 250 bp paired-end format.

### Sequence data processing and analysis

Raw reads (2,181,473 total) were processed using a mothur pipeline (v.1.44.1) to filter reads for quality, create contigs, and reduce noise (Schloss et al. 2009, Schloss 2020). We aligned sequences with the SILVA database (v.138) (Quast et al. 2013), and identified and removed chimeras using the “chimera.vsearch” command in mothur. We removed sequences from cloacal samples that were identical to sequences present in the negative controls. A mock community (reliable sample of known taxonomic composition) was not available for this study, so we were unable to evaluate the accuracy of our sequencing run. Reads were classified in mothur using a Bayesian classifier according to taxa (“classify.seqs” command) and we filtered mitochondrial and chloroplast sequences from these classifications using the “remove.lineage” command, which removed 9.2% of sequences. The SILVA database (version 138) was used to classify representative sequences and operational taxonomic units (OTUs) defined at an evolutionary distance of 0.03 (97% sequence similarity) using mothur's “opticlust” algorithm. Although the mothur package generates OTUs rather than amplicon sequence variants (ASVs), a consensus has not yet emerged on which approach better assesses host microbiome diversity and taxonomy. Our use of OTUs aligns with several recent studies of avian gut and cloacal microbiomes (e.g., Escallón et al. 2019, Murray et al. 2020, Capunitan et al. 2020). We removed samples with < 2000 sequences in R during statistical analyses, after which the number of retained reads per sample ranged from 2188 to 77,641 (*n* = 27 samples; average 22,056 reads/sample). Final sample sizes included in statistical analyses were as follows: pre-captivity = 18 (*n* = 6 neophobic, 12 non-neophobic; 5 females, 13 males) and post-captivity = 9 (*n* = 4 neophobic, 5 non-neophobic; 2 females, 7 males). Seven males were classified as neophobic, eight males were classified as non-neophobic, two females were classified as neophobic, and five females were classified as non-neophobic. Seven sparrows had pre- and post-captivity samples survive quality filtering (two neophobic, five non-neophobic).

Statistical analyses were conducted in R version 4.0.2 (R Core Team 2020). To avoid loss of power and decreased sensitivity (McMurdie and Holmes 2014), we focused on interpreting non-rarefied data. However, we have also reported rarefied results in the Supplementary Material to facilitate comparisons with studies that opt to rarefy their data. To assess differences in OTU diversity, non-parametric Shannon, Chao1, and inverse Simpson indices of alpha diversity were calculated using in mothur (“summary.single” command) once without subsampling and once with subsampling. To test the hypothesis that neophobic behavior affects gut microbiome alpha diversity, and that captivity may differentially affect microbiome alpha diversity of neophobia phenotypes, we conducted three linear mixed effect model that included subject ID as a random effect, sex as a main effect, and the interaction between neophobia phenotype and captivity (“lmer” command, (Kuznetsova et al. 2017)). These models differed in the measure of alpha diversity as the dependent variable (non-parametric Shannon, Chao1, and inverse Simpson). We performed pairwise comparisons using the “emmeans” command (Lenth 2021) to examine differences in phenotypes pre- and post-captivity, respectively, while controlling for the effect of sex. The sample size for post-captivity females was small (*n* = 2), so we only tested for sex effects in wild (pre-captivity) samples using three linear models. These models included only pre-captivity samples and differed in the dependent variable (non-parametric Shannon, Chao1, or inverse Simpson) and included only sex as a main effect. We visualized alpha diversity with phyloseq (McMurdie and Holmes 2013) and ggplot2 (Wickham et al. 2016) packages in R.

We computed Bray–Curtis dissimilarities using the “distance” function in phyloseq to test the hypothesis that neophobic behavior affects gut microbiome beta diversity, and that captivity may differentially affect microbiome alpha diversity of neophobia phenotypes with a permutational multivariate analysis of variance (PERMANOVA) using the “adonis” function from the vegan package (Oksanen et al. 2019) with 999 permutations with phenotype, captivity, and sex as main effects and the interaction between phenotype and captivity. To investigate natural (pre-captivity) differences between neophobia phenotypes and sexes, we subsetted the phyloseq object to contain only pre-captivity samples and performed an additional PERMANOVA test with sex and phenotype as factors. We tested for dispersion differences among groups using the “betadisper” function from the vegan package to confirm that mean tendency and dispersion differences among groups were not confounded. We used the phyloseq package to produce principal coordinates analysis (PCoA) ordinations with the “ordinate” function using Bray–Curtis and Jaccard dissimilarity, and to visualize the ordinations with the “plot_ordination” function. This was repeated upon a rarefied dataset that was produced using the “rarefy_even_depth” command in the phyloseq package.

We contrasted the relative abundance of phyla, family, and genera according to captive status, neophobia phenotype, and sex. Neophobia phenotype and sexes were examined for pre-captivity samples only. OTUs classified at 100% accuracy to a phylum in mothur were included in relative abundance analyses, and ≥80% accuracy for families and genera. We report phyla with relative abundance >5% in-text and a complete record available in Supplementary Material.

We used the multipatt function in the indicspecies package (Dufrêne and Legendre 1997, De Cáceres and Legendre 2009) to identify which OTUs were driving differences between captive status, neophobia phenotype, and sex. This analysis creates an indicator value for each OTU by computing the product of the relative abundance frequency of each OTU in the predefined groups. Significance of the relationship between OTUs among groups were based on permutation tests using 9999 random permutations to estimate *P*-values. Significant OTU comparisons were corrected with the false discovery rate procedure (Benjamini and Hochberg 1995) implemented in the “p.adjust” command in the stats package. OTU values with an indicator value > 0.5 and *P*-value < 0.05 were considered as indicator species (indicator value of 1 means the OTU is exclusive to one group). To visualize the abundance of indicator species, we created a heatmap using the pheatmap function of the pheatmap package (Kolde 2019). We tested for indicator OTUs in pre-captivity samples only for neophobia phenotype and sex. A rarefied dataset was produced in mothur without replacement using the subsample command for the rarefied indicator species analysis.

## Results

**Fig. 1:**
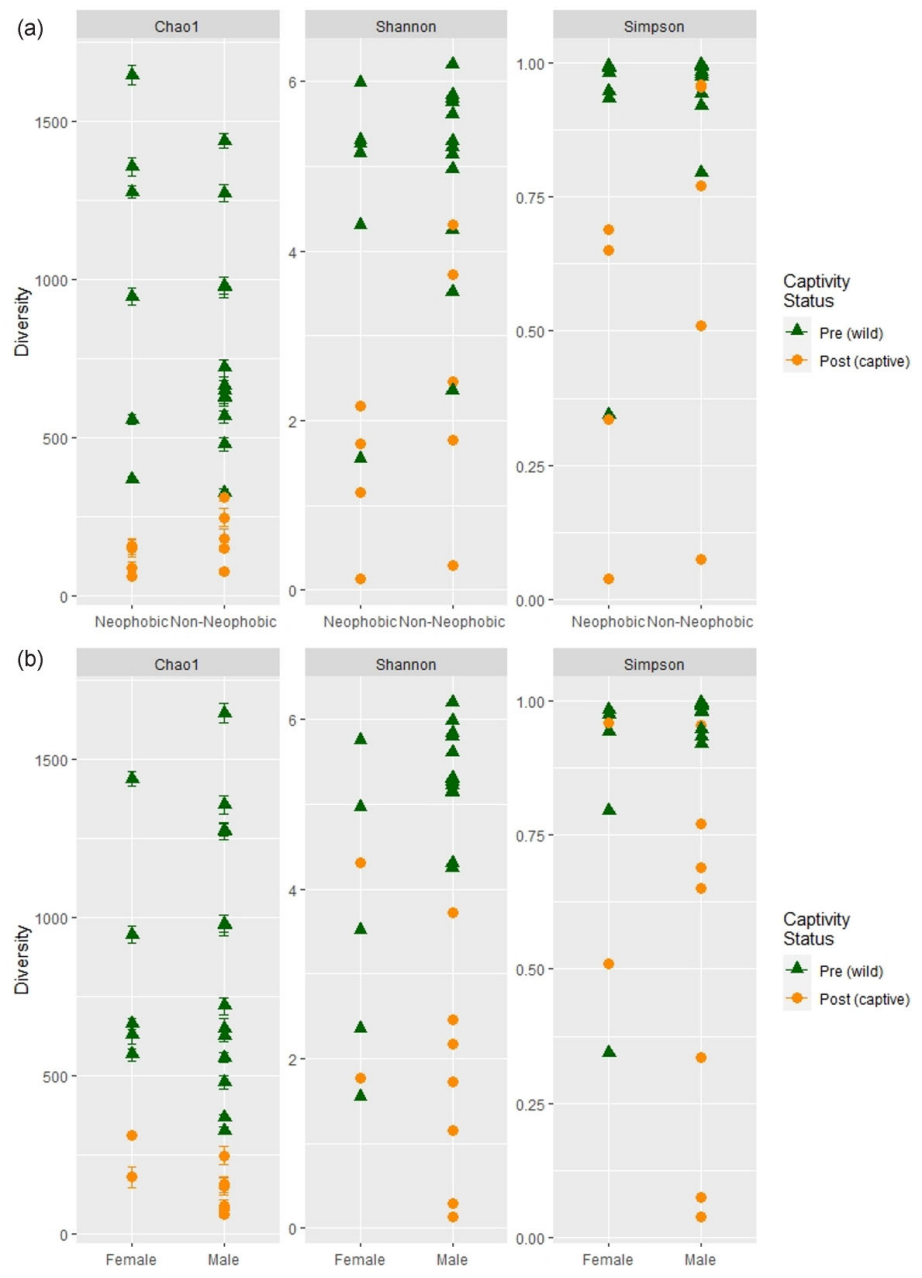
Non-rarefied alpha diversity decreased after eight weeks in captivity. Alpha diversity (Chao 1, non-parametric Shannon, and inverse Simpson) of house sparrow bacteria from cloacal swabs without rarefying data. House sparrows were sampled before (green triangles) and after (orange circles) exposure to captivity. **(a)** Neophobia phenotype. Final samples sizes were as follows: pre-captivity = 18 (n = 6 neophobic, 12 non-neophobic) and post-captivity = 9 (*n* = 4 neophobic, 5 non-neophobic). Pre-captivity samples had higher diversity than post-captivity samples (p < 0.01) but there was no effect of neophobia phenotype. **(b)** Sex. Final sample sizes were as follows: pre-captivity (wild) male = 13; post-captivity (captive) male = 7; pre-captivity (wild) female = 5; post-captivity (captive) female = 2. Males had significantly higher alpha diversity pre-captivity for non-parametric Shannon and inverse Simpson metrics. No contrasts were performed post captivity.

*Alpha diversity.* The mean Matthew's correlation coefficient (0.98) estimated high quality of OTU assignments of 16S rRNA sequences from house sparrow cloacal swabs. The resulting OTU table contained 7006 unique OTUs. OTU alpha diversity was higher in pre-captivity samples than post-captivity samples for all metrics investigated (non-parametric Shannon, Chao1, and inverse Simpson; all p ≤ 0.04) and this was true for both rarefied and non-rarefied data (Table 1a and S1a; Fig. 1a and S1a). Although we did detect a significant interaction between captivity and neophobia phenotype, the interaction was only significant for one estimate of alpha diversity (Chao1; Table 1b) and did not persist in rarefied analyses (Table S1a). In this interaction, both phenotypes exhibited a significant loss in Chao1 alpha diversity after captivity. Neophobic and non-neophobic sparrows did not differ in alpha diversity overall (Table 1a), nor when phenotypes were compared pre-captivity (neophobic house sparrows; non-parametric Shannon: t_22_ = −0.9, p = 0.35; Chao1: t_19.__2_ = 1.8, p = 0.08; inverse Simpson: t_21.4_ = −2.0, p = 0.06) or post-captivity (neophobic house sparrows; non-parametric Shannon: t_22_ = −1.9, p = 0.07; Chao1: t_21.9_ = −1.8, p = 0.09; inverse Simpson: t_21.4_ = −1.5, p = 0.1). Males had higher alpha diversity than females for two of the three alpha diversity metrics in non-rarefied and rarefied analysis (Table 1c, S1b; Fig. 1b). Rarefied results are reported in the Supplementary Material (Table S1; Fig. S1).

**Table 1:** Non-rarefied alpha diversity of house sparrow cloacal microbiomes was affected by captivity, but not neophobia phenotype. **(a)** Results of linear mixed effects models for the effects of captivity and neophobia phenotype on cloacal alpha diversity. Results are reported for three different alpha diversity metrics: non-parametric Shannon, Chao, and inverse Simpson. Final samples sizes were as follows: pre-captivity = 18 (*n* = 6 neophobic, 12 non-neophobic) and post-captivity = 9 (*n* = 4 neophobic, 5 non-neophobic). Captivity effects are estimated for pre-captivity samples, for non-neophobic house sparrows and for females. **(b)** Tukey post-hoc tests using estimated marginal means, extracted from the Chao linear mixed model in (a). **(c)** Results of linear models for the effect of sex on cloacal alpha diversity in wild (pre-captivity) samples. Effects are estimated for females (*n* = 5), with respect to males (*n* = 13). Statistically significant results are italicized.

	Parameter estimate ± standard error	df	t	p	95% confidence interval
**(a) overall *Non-parametric Shannon***					
*Captivity*	*3.5 ± 0.8*	*18.2*	*4.5*	*0.0003*	*2.0–4.9*
Phenotype	1.8 ± 0.8	22.0	2.1	0.05	0.1–3.4
Sex	−1.2 ± 0.6	16.4	−2.0	0.06	−2.3 to −0.1
captivity × phenotype	−1.2 ± 1.0	16.7	−1.1	0.3	−3.1–0.8
***Chao***					
*Captivity*	*1242.3 ± 128.2*	*4.5*	*9.0*	*0.0005*	*621.0–1509.8*
Phenotype	381.8 ± 208.2	21.8	1.8	0.08	−296.5–803.3
Sex	53.2 ± 195.3	13.5	0.3	0.8	−326.4–425.9
*captivity × phenotype*	*−714.3 ± 167.5*	*4.0*	*−4.3*	*0.01*	*−1030.8–24.5*
***Inverse Simpson***					
*Captivity*	*62.4 ± 27.4*	*13.8*	*2.3*	*0.04*	*621.1–1509.8*
Phenotype	49.2 ± 31.1	21.3	1.6	0.1	−296.53–803.3
*Sex*	*−64.4 ± 22.5*	*14.6*	*−2.9*	*0.01*	*−326.5–425.9*
captivity × phenotype	−3.2 ± 34.9	11.4	−0.1	0.9	−1030.8–24.5
**(b) post-hoc: Chao, captivity × phenotype**					
pre-captivity: neophobic (non-neo)	332 ± 182	19.2	1.8	0.08	
post-captivity: neophobic (non-neo)	−382 ± 212	21.9	−1.8	0.08	
*neophobic: post-captivity (pre)*	*−1242 ± 148.1*	*7.4*	*−8.4*	*<0.001*	
*non-neophobic: post-captivity (pre)*	*−528 ± 96.8*	*5.6*	*−5.5*	*0.002*	
**(c) wild (pre-captivity) *Non-parametric Shannon***					
*Sex*	*−1.7 ± 0.5*	*16*	*−3.2*	*0.005*	*−2.9 to −0.6*
***Chao***					
Sex	−15.6 ± 213.8	16	−0.07	0.9	−468.8–437.6
***Inverse Simpson***					
*Sex*	*−72.0*	*16*	*−2.5*	*0.02*	*−133.0 to −10.9*

**Fig. 2:**
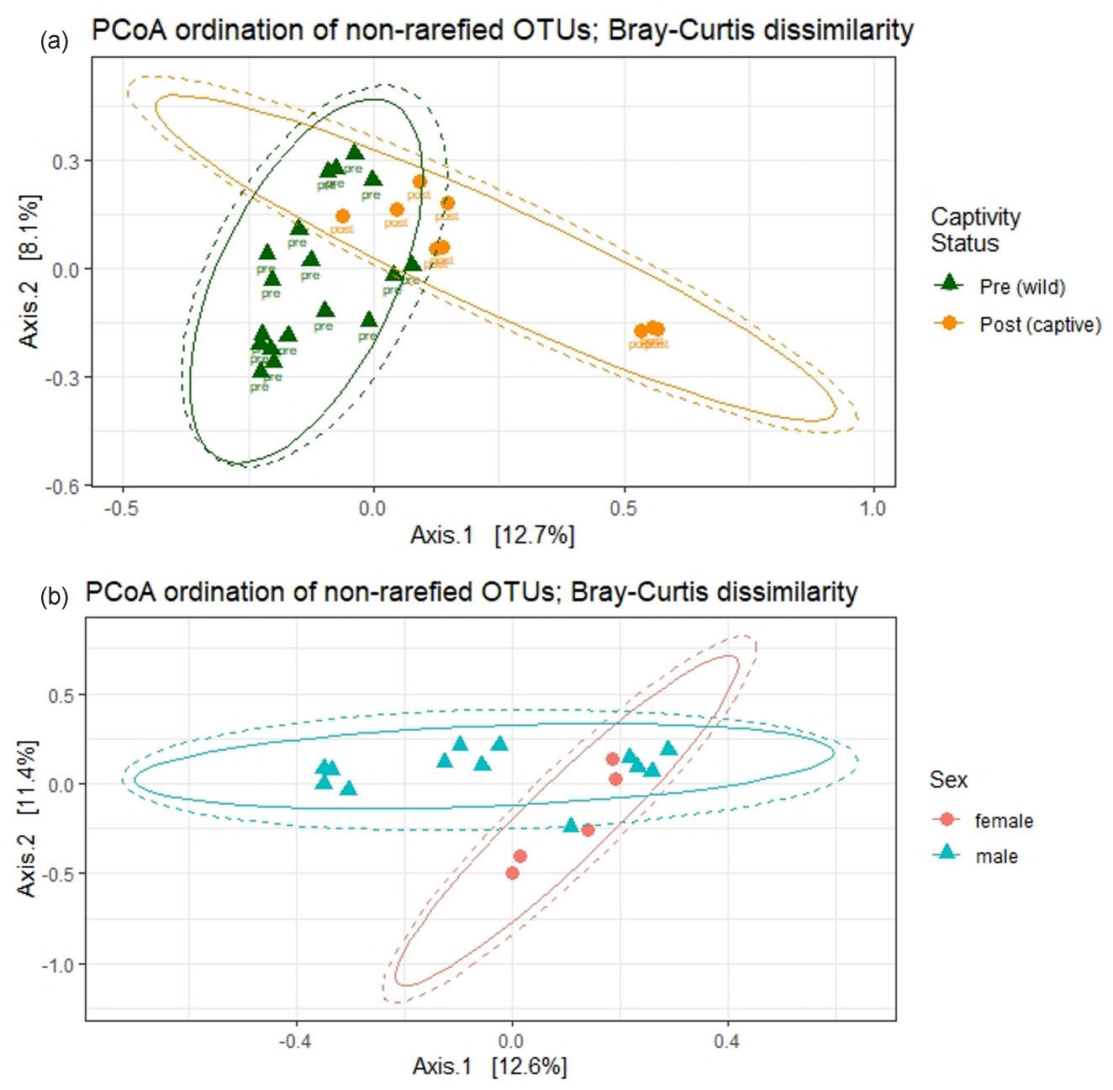
Non-rarefied cloacal community composition differed between wild and captive states. Principal coordinates analysis using a Bray–Curtis dissimilarity matrix of OTUs illustrates the shift between **(a)** pre- (green triangles; *n* = 18) and post-captivity (orange circles; *n* = 9) and **(b)** the difference between wild male (blue triangles; *n* = 13) and wild female (pink circles; *n* = 5) bacteria community composition in cloaca swabs. No difference was detected between neophobic and non-neophobic phenotypes (Fig. S2). Each point represents a cloacal sample from an individual bird. Increasing distance between points indicates increasing dissimilarity in cloacal community composition. The visualization using Jaccard distances is similar and thus not reported but can be produced using R code available in the Supplementary Material.

*Beta diversity.* No dispersion differences were detected among groups considering all samples for Bray–Curtis distances (captivity: F_1,__25_ = 0.6, p = 0.4; neophobia phenotype: F_1,__25_ = 0.2, p = 0.7; sex: F_1,__25_ = 0.5, p = 0.5) or for Jaccard distances (captivity: F_1,__25_ = 0.01, p = 0.9; neophobia phenotype: F_1,__25_ = 0.05, p = 0.8; sex: F_1,__25_ = 3.6, p = 0.08). Considering only pre-captivity samples, no dispersion differences were detected between neophobia phenotypes (Bray–Curtis: F_1,__16_ = 1.0, p = 0.3; Jaccard: F_1,__16_ = 3.3, p = 0.09) or between sexes (Bray–Curtis: F_1,__16_ = 0.1, p = 0.7; Jaccard: F_1,__16_ = 2.3, p = 0.1). The first three axes resulting from the PCoA analysis using Bray–Curtis and Jaccard dissimilarity accounted for 28.0 and 21.5%, respectively, of the variability in the entire dataset; 31.6 and 25.3%, respectively, for pre-captivity samples only (Table S2). The PERMANOVA test including all samples did not detect differences in the community composition of house sparrow cloaca bacteria between neophobia phenotypes, nor an interaction with captivity, but did detect a difference between pre-captivity samples and post-captivity samples for Bray–Curtis and Jaccard distances on both rarefied and non-rarefied data (Table 2a, S3a; Fig. 2a). The PERMANOVA test upon pre-captivity samples detected a significant sex difference in the community composition of wild house sparrow cloaca microbiomes for both Bray–Curtis and Jaccard distances upon rarefied and non-rarefied data (Table 2b, S3b; Fig. 2b), but no differences between phenotypes. Rarefied results are reported in the Supplementary Material (Table S3).

**Table 2:** Non-rarefied beta diversity of house sparrow cloacal microbiomes was affected by captivity, but not neophobia phenotype. **(a)** Results of permutational multivariate analysis of variance (PERMANOVA) tests using Bray–Curtis and Jaccard dissimilarity that tested for effects of captivity, neophobia, and their interaction upon beta diversity. Each factor had a similar dispersion. Final samples sizes were as follows: pre-captivity = 18 (*n* = 6 neophobic, 12 non-neophobic) and post-captivity = 9 (n = 4 neophobic, 5 non-neophobic). **(b)** Results of PERMANOVA tests using Bray–Curtis and Jaccard dissimilarity that tested for the effect of sex and phenotype upon beta diversity in wild (pre-captivity) samples (*n* = 13 males, 5 females). Abbreviations: SS, sum of squares; MSS, mean sum of squares; F, F statistic. Rarefied analyses are reported in Table S2 of the Supplementary Material. Statistically significant results are italicized.

	**Bray–Curtis** Fig. 2a					Jaccard				
	df	SS	MSS	F	p	df	SS	MSS	F	p
**(a)**										
*captivity*	*1*	*1.0*	*1.0*	*2.5*	*0.001*	*1*	*0.8*	*0.8*	*1.8*	*0.001*
phenotype	1	0.4	0.4	1.1	0.3	1	0.4	0.4	1.0	0.4
sex	1	0.5	0.5	1.1	0.2	1	0.5	0.5	1.1	0.2
captivity × phenotype	1	0.4	0.4	1.1	0.2	1	0.5	0.5	1.0	0.3
residuals	22	8.8	0.4			22	9.7	0.4		
total	26	11.1				26	11.9			
**(b)**										
*sex*	*1*	*0.5*	*0.5*	*1.4*	*0.02*	*1*	*0.5*	*0.5*	*1.2*	*0.03*
phenotype	1	0.4	0.4	1.2	0.1	1	0.5	0.5	1.1	0.1
residuals	15	5.6	0.4			15	6.4	0.4		
total	17	6.6				17	7.4			

**Fig. 3:**
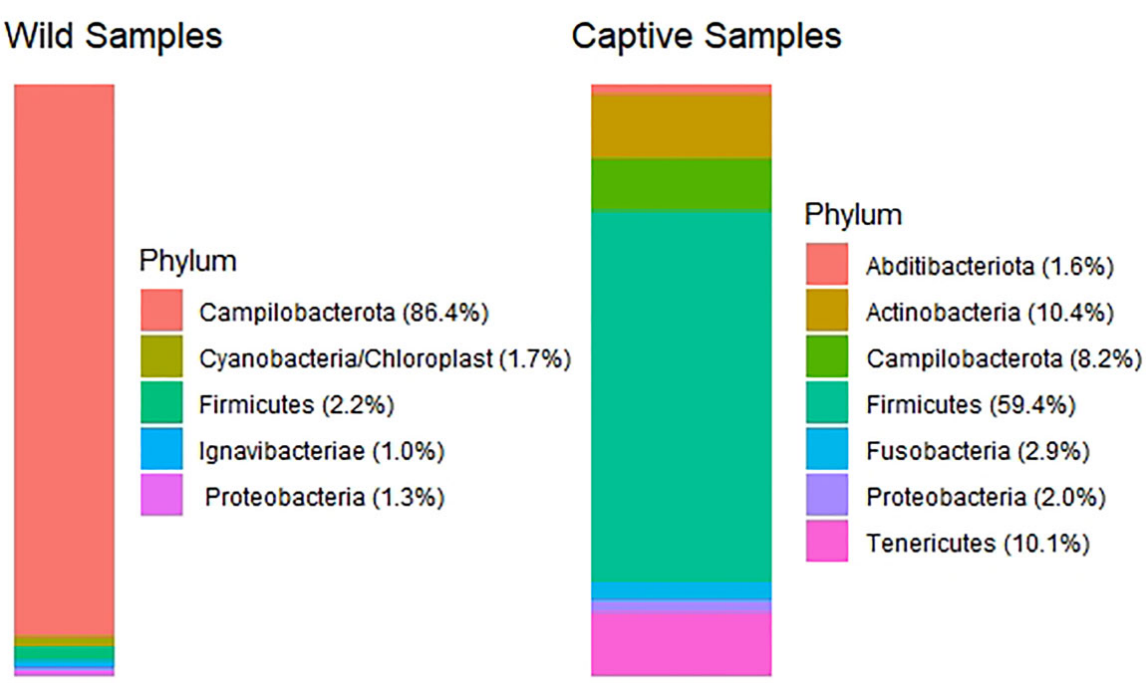
Top (>1%) phyla of cloacal bacteria differed pre- and post-captivity. Non-rarefied abundance of dominant (>1% relative abundance) bacteria phyla present in cloacal samples from house sparrows, pre- (wild; *n* = 18) and post-captivity (captive; *n* = 9).

*Relative abundance.* With respect to all the OTUs classified at 100% accuracy to a phylum in mothur, the relative abundance of dominant bacterial phyla (>5%) in neophobic sparrows only included Campilobacterota (94%); Campilobacterota was also the top bacterial phylum in non-neophobic sparrow cloacae, but at lower abundance (14.6%), followed by Firmicutes (14%), Ignavibacteriae (10.9%), Proteobacteria (8.6%), Latescibacteria (6.2%), and Actinobacteria (6%; Fig. S3). Campilobacterota had the highest relative abundance for pre-captivity samples (86.4%) and no other phyla had relative abundance >5% (Fig. 3). Firmicutes (59.4%) had the highest relative abundance in post-captivity samples, followed by Actinobacteria (10.4%), Tenericutes (10.1%), and Campilobacterota (8.2%; Fig. 3). Female cloacae were dominated by Campilobacterota (95.4%). Campilobacterota was also the top bacterial phyla in male sparrow cloacae (38.1%), followed by Cyanobacteria/Chloroplast (9.2%), Firmicutes (6.7%), and Proteobacteria (5.7%). Complete relative abundance of phyla, families, and genera for neophobia phenotype, captivity, and sex are reported as supplementary Excel files upon rarefied and non-rarefied data.

**Fig. 4:**
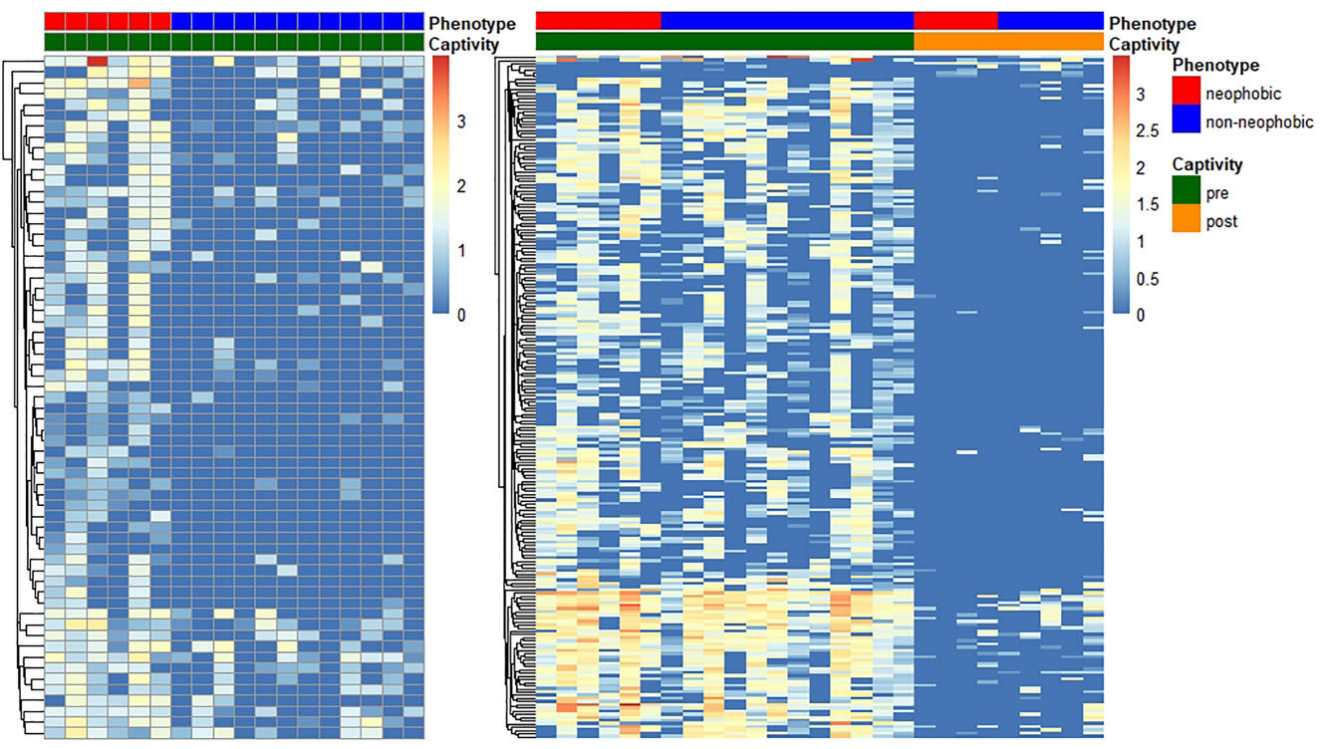
Cloacal indicator OTUs. Heat map of the relative abundances of non-rarefied indicator OTUs from indicator species analysis associated with neophobic house sparrow microbiomes pre-captivity (left; *n* = 78), and indicator OTUs associated with captivity status (right; pre-captivity *n* = 210, post-captivity *n* = 5). No indicator OTUs were associated with non-neophobic house sparrow cloacae. Rows indicate unique OTUs and columns individual birds. The lowest taxonomic resolution that could be defined for OTU identification is listed in an Excel file (see Supplementary Material), as are indicator values, *P* values, FDR-corrected *P* values, positive predictive values, and sensitivity values. Heatmap of rarefied results are available in the Supplementary Material (Fig. S3).

*Indicator species analysis.* Neophobia indicator species analysis of non-rarefied pre-captivity data revealed 78 indicator OTUs associated with the neophobic house sparrow cloacal microbiome and none associated with non-neophobic sparrows (Fig. 4). Relative abundance of neophobic indicator OTUs by phyla were 43.7% Cyanobacteria/Chloroplast, 26% Firmicutes, and 7.9% Actinobacteria. Of the 41 families represented by these neophobic indicator OTUs, Alicyclobacillaceae had the highest relative abundance (62%), followed by Cyanobacteria/Chloroplast (8.2%). Of the 47 genera represented, *Tumebacillus* had the highest relative abundance (55.9%), followed by unclassified *Cyanobacteria*/Chloroplast (7.4%).

Captivity indicator species analysis of non-rarefied data revealed 210 OTUs associated with pre-captivity cloacal swab samples and 5 associated with post-captivity (Fig. 4). The relative abundance of significant pre-captivity indicator OTUs by phyla included Cyanobacteria/Chloroplast (52.6%), Actinobacteria (10.9%), Acidobacteria (7.5%), Firmicutes (6.5%), Bacteroidetes (5.6%), Proteobacteria (5.3%), and Verrucomicrobia (5.1%). Of the 75 families, unclassified Cyanobacteria/Chloroplast had the highest relative abundance (8.2%), followed by Enterobacteriaceae (6.6%), unclassified Rhizobiales (6.4%), and Corynebacteriaceae (5.2%). Of the 101 genera, unclassified Cyanobacteria/Chloroplast had the highest relative abundance (6.4%), followed by *Klebsiella* (5.2%). The relative abundance of significant post-captivity indicator OTUs by phyla included Proteobacteria (55%) and Firmicutes (43%); for family (*n* = 4), Sphingomonadaceae (61%), Streptococcaceae (27.7%), and Pseudomonadaceae (9.9%); for genus (*n* = 4), *Sphingomonas* (61.1%), *Streptococcus* (27.7%), and *Pseudomonas* (9.9%).

Indicator species analysis of sex upon non-rarefied data revealed 28 OTUs associated with pre-captivity female cloacal swab samples, and none associated with males. The relative abundance of phyla for female indicator OTUs was primarily Campilobacteria (93.8%) followed by Firmicutes (5.5%). Of the 22 families present, Campylobacteraceae had the highest relative abundance (77.5%), followed by Enterococcaceae (15.8%). Of the 24 genera present, 77.4% of their relative abundance was *Campylobacter*, followed by *Enterococcaceae* (15.7%). For all indicator species analysis, a list of all statistically significant OTUs, their indicator values, FDR- corrected *P*-values, positive predictive values, sensitivity values, and their taxonomic classification for rarefied and non-rarefied data is available as a supplementary Excel file (see Supplementary Material). The relative abundance of all phyla, families, and genera for indicator OTUs (rarefied and non-rarefied) are reported in a supplementary Excel file.

## Discussion

Several different factors have been shown to affect the composition of the gut and cloacal microbiome in birds, including sex, age, diet, and genetics (Mills et al. 1999, Lumpkins et al. 2008, van Dongen et al. 2013, Zhao et al. 2013, Ballou et al. 2016, Barbosa et al. 2016, Pearce et al. 2017, Kohl et al. 2018), though few specific links between avian behavioral traits and the microbiome have been found (but see (Davidson et al. 2020b)). In mammals, early life acquisition of microbes (or lack thereof) can affect the development of the central nervous system and its function, which is reflected in the behavior of germ-free mice (Neufeld et al. 2011a,b). For example, eventual colonization of gut microbiota in adulthood influences adult exploratory and anxiety behaviors (Bercik et al. 2011, Clarke et al. 2013). The sources of the microbes in the avian gut include regurgitation from parents in altricial species (Kyle and Kyle 1990, Godoy-Vitorino et al. 2010), the diet (Maul et al. 2005, Hird et al. 2014, Waite and Taylor 2014), and the environment (Lucas and Heeb 2005, van Dongen et al. 2013, Hird et al. 2014, Barbosa et al. 2016, van Veelen et al. 2017) although, to our knowledge, no studies have sampled the same individuals at multiple time points to examine whether nestling microbiomes are maintained throughout adulthood.

If the adult gut microbiome were one of the driving forces behind variation in neophobia behavior in wild-caught house sparrows, we should have detected differences in alpha and beta diversity of the cloacal microbiomes of neophobic and non-neophobic sparrows. However, contrary to our predictions, we saw no differences in microbiome diversity between the two phenotypes, suggesting adult microbiome diversity does not mediate neophobia in this species. Alternatively, a relationship between neophobia and the gut microbiome may occur at the level of specific taxa, or taxa that exert effects at low abundances. Indeed, we did detect a higher frequency and abundance of 78 specific OTUs associated with neophobic sparrows using an indicator species analysis and none associated with non-neophobic sparrows. The relative abundance of the neophobic indicator OTUs were primarily Tumebacillus genera (55.9%), in the family Alicyclobacillaceae (62%). Alicyclobacillaceae was identified as a dominant family in the gut of *Rhopalotria furfuraceae* (Coleoptera; beetle) and *Luthrodes pandava* (Lepidoptera; butterfly) that feed on carcinogenic and neurotoxic tissues of cyad plants (Salzman et al. 2018). Tumebacillus has also been isolated from the gut of a cinereous vulture (*Aegypius monachus*) in South Korea (Sung et al. 2018). However, Alicyclobacillaceae and Tumebacillus have also been isolated from diverse environments including permafrost in Canadian high Arctic (Steven et al. 2008), soil in South Korea (Baek et al. 2011) and Ukraine (Her et al. 2015), cassava wastewater in southern China (Wang et al. 2013), decomposing algal scum in China (Wu et al. 2015), and river water in India (Prasad et al. 2015). Whether Alicyclobacillaceae and Tumebacillus are present in the sparrow gut due to ingestion or an evolutionarily conserved functional role in the gut (e.g., some species of Tumebacillus can produce amylase (Wang et al. 2013)) is yet to be determined and warrants future functional study. Indicator species analysis is becoming a valuable tool in microbial ecology and has revealed specific microbes associated with traits such as age and breeding status in rufous-collared sparrows (*Zonotrichia capensis*) (Escallón et al. 2019) and survival in nestling great tits (*Parus major*) (Davidson et al. 2021).

Low sample sizes after quality filtering of data might have limited our ability to detect true differences in microbial diversity between neophobic and non-neophobic sparrows. The only other study to our knowledge that tests for a relationship between a behavioral trait and gut microbiome diversity in a wild avian species also found no relationship between fecal microbiome diversity and the ability to solve a novel foraging task in captivity, despite sampling a larger number of birds (*n* = 36 (Davidson et al. 2020b)). Intriguingly, however, a relationship was seen between diversity of the fecal microbiome and foraging innovation after a captive dietary manipulation. Thus, although there are undeniable impacts of the gut microbiome on the brain in humans and lab-reared species like mice, there is a clear need to better understand this relationship in wild species. For example, this species’ social learning ability could have impacted our findings such that the aversion to novelty we detected in the lab may not meaningfully impact food choices in *flocks* of wild sparrows. House sparrows are gregarious in the wild (Lowther and Cink 2020) and neophobic sparrows can learn from conspecifics to be less neophobic (Kelly et al. 2020); thus, although neophobic sparrows may not be the first in their flock to eat a novel food, it is possible that they do eventually eat and explore novel items, leading to similar gut microbiomes as non-neophobic individuals. A final possibility is that the *nestling* microbiome could contribute to a neophobic phenotype, even if it is not retained into adulthood. This possibility highlights the need for studies that manipulate the microbiome during development to conclusively assess how the microbiome may affect personality traits in wild species. Meanwhile, our study does not support the hypothesis that the adult gut microbiome influences neophobia in house sparrows.

Regardless of neophobia phenotype, exposure to captivity caused a significant reduction in house sparrow cloacal microbiome alpha diversity, consistent with results in other bird species (Xenoulis et al. 2010, Wienemann et al. 2011, Ushida et al. 2016). Captivity also caused a shift in cloacal microbiome community composition (beta diversity), similar to results in rock ptarmigan (*Lagopus muta*) (Ushida et al. 2016, Salgado-Flores et al. 2019). The biggest shift in phyla upon exposure to captivity was the loss of Campilobacterota with 86% relative abundance in wild samples compared to 8% in captive samples. This result was driven by neophobic sparrows (94% abundance compared to 14.6% in non-neophobic sparrows) and females (95% abundance, compared to 38% abundance in males). Indicator OTUs of neophobic sparrows did not include Campilobacterota, but the relative abundance of female indicator OTUs were 94% Campilobacterota. Only one OTU (Genus: *Campylobacter*) was represented by the Campilobacterota phylum in female indicator species analysis. BLAST results of the sequence corresponding to this OTU was inconclusive and returned 99.2% identity to *Campylobacter armoricus*, *C. aviculae*, *C. taeniopygiae*, and *C. novaezeelandiae* (accession # CP053825.1, MK458937.1, MK458935.1, and CP076657.1). Further investigation revealed that this relationship was driven by a single individual. *Campylobacter* causes no clinical disease in adult poultry (Shane 2000) and shedding is highest in the summer months (Colles et al. 2009), so this sparrow likely had an active infection at capture, resulting in very high abundance of this bacteria. *Campylobacter* is often carried by house sparrows (Benskin et al. 2009) and European starlings (*Sturnus vulgaris* (Colles et al. 2009). These results are also in line with other research in house sparrows, in which wild sparrows were most distinguished by taxa from the genus *Campylobacter* compared to captive sparrows (Madden et al., in press), suggesting that captivity may reduce some potentially pathogenic bacteria.

At the genus level, 657 genera were detected in wild samples and only 234 in captive samples. In line with this, we identified 210 OTUs that were found more frequently and at higher abundances in pre-captivity cloacal samples than in post-captivity samples, and 5 associated with post-captivity. This marked loss of genera (64%) in captivity is likely in part because of the increased relative abundance of Firmicutes and, to a lesser extent, Actinobacteria, both of which have important functional roles in the gut. Many animals benefit from Actinobacteria to help digest complex plant-derived materials (Lewin et al. 2016). Firmicutes produce short-chain fatty acids as byproducts of carbohydrate metabolism to be absorbed by the host (Den Besten et al. 2013), and a high abundance of Firmicutes has been linked to weight gain in chickens (Angelakis and Raoult 2010). Although house sparrows in this study did not gain weight in captivity, previous work in house sparrows has shown that captivity causes major shifts in body composition, increasing fat volume and decreasing muscle density (Lattin et al. 2017). Captivity can also cause increased baseline corticosterone concentrations in wild songbirds that can persist for weeks (Marra et al. 1995, Lattin et al. 2012, Love et al. 2017), and increases in corticosterone have been shown to alter the gut microbiome in wild birds (Noguera et al. 2018).

Another potential reason for the shift in the microbiome could be due to the mixed seed and Mazuri diet given to house sparrows, the latter of which included live microorganisms (complete list in Supplementary Material), including *Lactobacillus acidophilous*, *Lactobacillus casei*, and *Enterococcus faecium*, all Firmicutes, as well as *Bifidobacterium thermophilum*, an Actinobacterium. The Mazuri diet also contained ground corn, wheat middlings, and soybean meal. In another study, captive house sparrows given diets that contained similar foods as Mazuri diet to simulate urban (corn, bread, cake, and potato chips) and rural (corn, wheat, sunflower seed, mealworm) diets exhibited increases of *Lactobacillaceae* and *Enterococcaceae* in the cloaca compared to pre-captivity samples (Teyssier et al. 2020) similar to the increases we observed in the house sparrow microbiomes after captivity. Poultry given a mixed wheat and rye diet increased gram positive Enterococci in the gut compared to poultry given a pure corn diet (Hübener et al. 2002). The increase in Firmicutes microbiota observed among these studies may be because these taxa thrive on grains included in captive diets. Therefore, the increase in relative abundance of Firmicutes and Actinobacteria in captive sparrows appears to be a combined result of (1) losing a large number of wild-sourced genera whose disappearance artificially inflates the abundance of other microbes, and (2) gaining microbes from the captive diet. This provides further evidence that captive gut microbiome samples are not representative of gut microbial communities in free-living animals, although it seems likely that a diet including probiotics may have been responsible for some of the changes we saw in the captive microbiome.

We also found higher cloacal microbial diversity in male sparrows than females, different community compositions, and 28 OTUs specifically associated with females. Although some studies reveal sex differences in the cloaca or fecal microbiome of birds (Escallón et al. 2019, Liu et al. 2020), other studies do not (Kreisinger et al. 2015, Corl et al. 2020, Góngora et al. 2021). There may be some aspect of a species’ life history that explains these patterns—for example, dietary differences between sexes, or sex differences in the frequency of extra-pair copulations—and this bears further investigation. In summary, although there are clear links between the gut microbiome and the behavior of laboratory mice (Neufeld et al. 2011b, Foster and McVey Neufeld 2013) and humans (Benton et al. 2007, Rao et al. 2009, Messaoudi et al. 2011), the extent to which these relationships exist in wild animals is largely unknown (Davidson et al. 2020a). If the adult gut microbiome were an important mediator of neophobia in house sparrows, we should have detected distinct differences in cloacal microbial diversity in neophobic and non-neophobic sparrows, and we did not. However, the identification of specific OTUs associated with higher abundance and detection frequency in samples from neophobic sparrows suggests possible relationships between personality and the gut microbiome at the level of specific taxa. Congruent with other studies, captivity reduced diversity and changed the composition of the cloacal microbiome similarly in neophobic and non-neophobic individuals. We strongly encourage further investigations of personality and the gut microbiome in more wild species to reveal how the microbiome-gut-brain axis and behavior interact in an ecological context.

## Supplementary Material

obac010_Supplemental_Files

## Data Availability

Data and R Code are available as Supplementary Material. The sequence data underlying this article are available in NCBI Sequence Read Archive (SRA) at https://www.ncbi.nlm.nih.gov/sra, and can be accessed with accession #SUB9595519.
