# Supplementary material for: No Guts About It: Captivity, But Not Neophobia Phenotype, Influences the Cloacal Microbiome of House Sparrows (Passer domesticus)
Source: Integr Org Biol. 2022 Mar 11;4(1):obac010. doi: 10.1093/iob/obac010 (PMC9053947; doi:10.1093/iob/obac010)
Supplement: obac010_Supplemental_Files [file obac010_supplemental_files.zip › 2022-02-10_Supplementary Material_v6_no markup_anonymous.docx]

**Supplementary Materials for:**

**No guts about it: captivity, but not neophobia phenotype, influences cloaca microbiome of
house sparrows (*Passer domesticus*)**

*Authors, institution and author correspondence omitted for double blind review.*

**Guide to Supplementary Files**

Files used to create the phyloseq object in R:
“R_OTU matrix.xlsx” (OTU abundance data)
“R_sample_data.xlsx” (Sample metadata)

- Abundance and metadata files are required for indicator species analysis.

“R_taxonomy.xlsx” (Taxonomy file)

- All three files are required for beta diversity R Code and relative abundance analyses.

Alpha Diversity:
“R Code_alpha diversity tests_not rarefied_R1.R” (R Code, non-rarefied)
“R_alpha diversity_non-rarefied.xlsx” (Data, non-rarefied)

“R Code_alpha diversity tests_rarefied_R1.R” (R Code, rarefied)
“R_alpha diversity_rarefied.xlsx” (Data, rarefied)

- Shapiro tests for normality, Bartlett’s test for equal variances.
- Linear mixed models for captivity-phenotype interactions.
- Linear models for sex effects.
- Visualization of Chao1, Shannon, and Simpson indices.

Beta Diversity:
“R Code_beta diversity tests_not rarefied_R1.R” (R Code, non-rarefied)
“R Code_beta diversity tests_rarefied_R1.R” (R Code, rarefied)

- Uses phyloseq object.
- Bray-Curtis dissimilarity matrix creation, test for homogeneity of multivariate dispersion (Levene’s test), and permutational multivariate analysis of variance (PERMANOVA).
- PCoA ordination & visualizations of ordinations.
- Rarefied visualizations are not reported but can be produced with the ‘rarefied’ R Code.

Indicator Species Analysis:
“R Code_indicator species_non-rarefied_R1.R” (R Code, non-rarefied)
“R_ indicator species_non rarefied.xlsx” (Abundance & sample data)
“R Code_indicator species_rarefied_R1.R” (R Code, rarefied)
“R_ indicator species_rarefied.xlsx” (Abundance & sample data)

- Indicator species analysis contrasting neophobia phenotypes of wild house sparrow cloaca microbiome samples, contrasting wild and captive samples, and sex differences in wild house sparrow cloaca microbiome samples.
- Heatmap visualizations of indicator species abundance, including only OTUs with an indicator value > 0.49 and *p* value < 0.05.

Indicator Species Results:
“Indicator Species Summary_2021-12-14.xlsx” (Results summary)

- Includes Phyla, Family, Genus, indicator value, raw *p* value, false discovery rate-corrected (FDR) *p* value, positive predictive value (A), and sensitivity value (B) of OTUs significantly associated with the cloaca microbiome of wild neophobic sparrows, wild and captive house sparrow cloaca microbiome, and wild female and male house sparrow cloaca microbiomes.
- Rarefied and non-rarefied analyses.

Relative Abundance of Indicator Species:

“R Code_relative abundance_indicator species.R” (R Code)

- Requires list of significant OTUs produced in Indicator Species Analysis

"Abundance summary_Indicator OTUs_Phyla_2022-01-14.xlsx” (Results summary)

- Relative abundance of phyla for significant indicator OTUs significantly associated with the cloaca microbiome of wild neophobic sparrows, wild and captive house sparrow cloaca microbiome, and wild female and wild male house sparrow cloaca microbiomes.
- Rarefied and non-rarefied analyses.

“Abundance summary_Indicator OTUs_Family_2022-01-14.xlsx” (Results summary)

- Relative abundance of families for significant indicator OTUs significantly associated with the cloaca microbiome of wild neophobic sparrows, wild and captive house sparrow cloaca microbiome, and wild female and wild male house sparrow cloaca microbiomes.
- Rarefied and non-rarefied analyses.

“Abundance summary_Indicator OTUs_Genus_2022-01-14.xlsx” (Results summary)

- Relative abundance of genera for significant indicator OTUs significantly associated with the cloaca microbiome of wild neophobic sparrows, wild and captive house sparrow cloaca microbiome, and wild female and wild male house sparrow cloaca microbiomes.
- Rarefied and non-rarefied analyses.

Relative Abundance of Phyla:
“R Code_relative abundance _phyla100_R1.R” (R Code)

- Uses phyloseq object and phyla classified at 100 % accuracy. Accuracy is generally high at this level and anything lower are likely problematic or novel sequences.

“Abundance summary_Phyla_2021-12-09.xlsx” (Results summary)

- Phyla relative abundance for wild versus captive samples, wild neophobic and non-neophobic samples, and wild male and female house sparrow samples.
- Rarefied and non-rarefied analyses.

Relative Abundance of Families:
“R Code_relative abundance_family80_R1.R” (R Code)

- Uses phyloseq object and families classified at 80% accuracy or higher.

“Abundance summary_Family_2022-01-17.xlsx” (Results summary)

- Family relative abundance for wild versus captive samples, wild neophobic and non-neophobic samples, and wild male and female house sparrow samples.
- Rarefied and non-rarefied analyses.

Relative Abundance of Genera:
“R Code_relative abundance_genus80_R1.R” (R Code)

- Uses phyloseq object. Genera classified at 80% accuracy or higher.

“Abundance summary_Genus_2022-01-17.xlsx” (Results summary)

- Genera relative abundance for wild versus captive samples, wild neophobic and non-neophobic samples, and wild male and female house sparrow samples.
- Rarefied and non-rarefied analyses.

**Supplementary Methods:**

**Mazuri® Small Bird Diet list of ingredients:**

Ground Corn, Wheat Middlings, Dehulled Soybean Meal, Corn Gluten Meal, Ground Flaxseed, Glyceryl Monostearate, Dicalcium Phosphate, Soybean Oil, Calcium Carbonate, Wheat Germ, Brewers Dried Yeast, Calcium Propionate (a preservative), Salt, L-Lysine, DL-Methionine, Dried ***Lactobacillus acidophilus*** (Firmicutes) Fermentation Product, L-Ascorbyl-2-Polyphosphate (Vitamin C), Choline Chloride, Dried ***Lactobacillus casei*** (Firmicutes) Fermentation Product, Dried ***Bifidobacterium thermophilum*** (Actinobacteria) Fermentation Product, Pyridoxine Hydrochloride, Biotin, Dried ***Enterococcus faecium*** (Firmicutes) Fermentation Product, d-Alpha Tocopheryl Acetate (Vitamin E), Menadione Sodium Bisulfite Complex (Vitamin K), Cholecalciferol (Vitamin D3), Preserved with Mixed Tocopherols, Rosemary Extract, Folic Acid, Vitamin A Acetate, Citric Acid (a Preservative), Riboflavin Supplement, Manganous Oxide, Zinc Oxide, Calcium Iodate, Calcium Pantothenate, Thiamine Mononitrate, Beta Carotene, Nicotinic Acid, Vitamin B12 Supplement, Copper Sulfate, L-Tryptophan, Zinc Sulfate, Sodium Selenite, Cobalt Carbonate.

Link: <https://info.mazuri.com/BusinessLink/media/Mazuri/ProductSheet/56A6.pdf>

**Supplementary Figures**
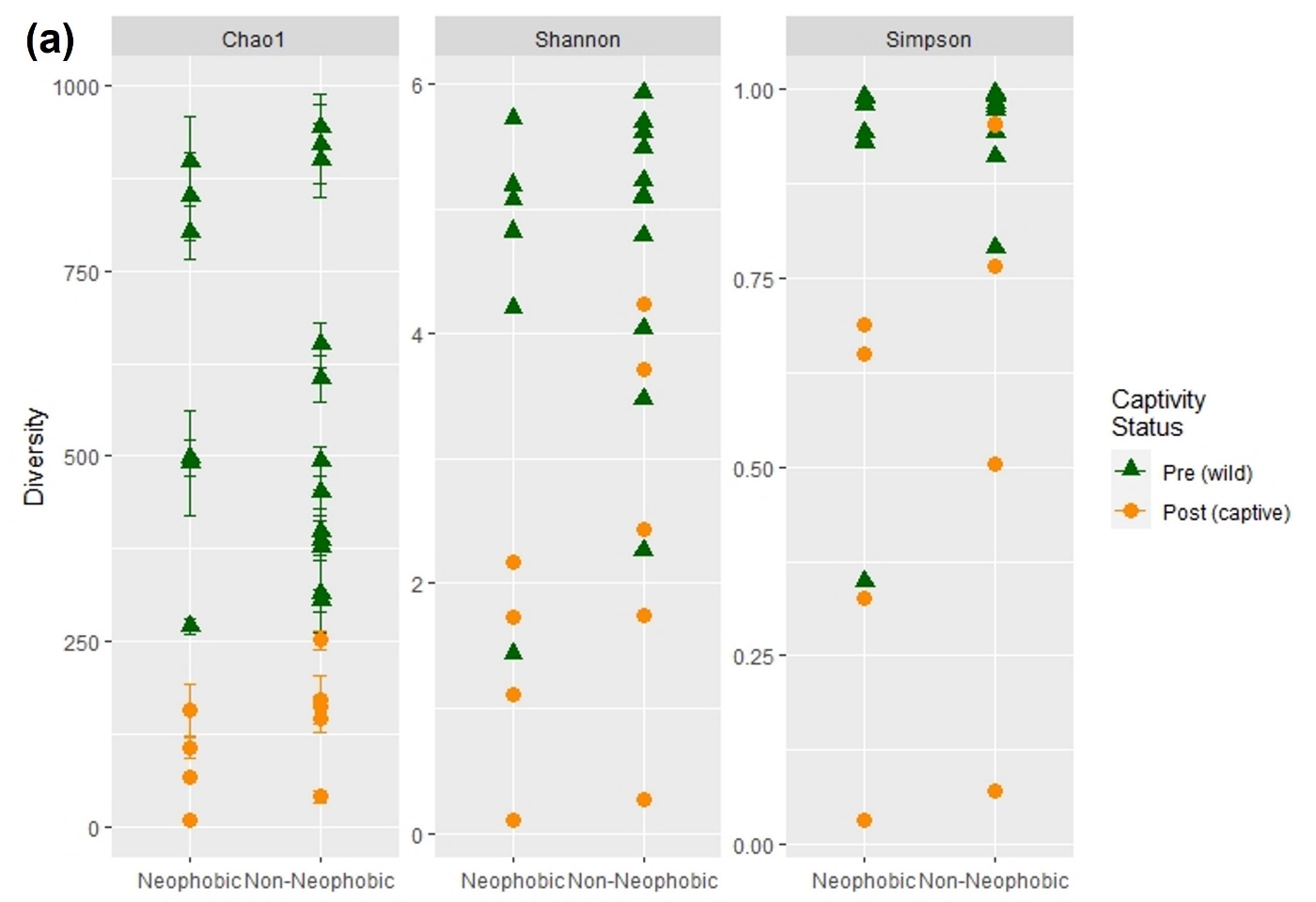

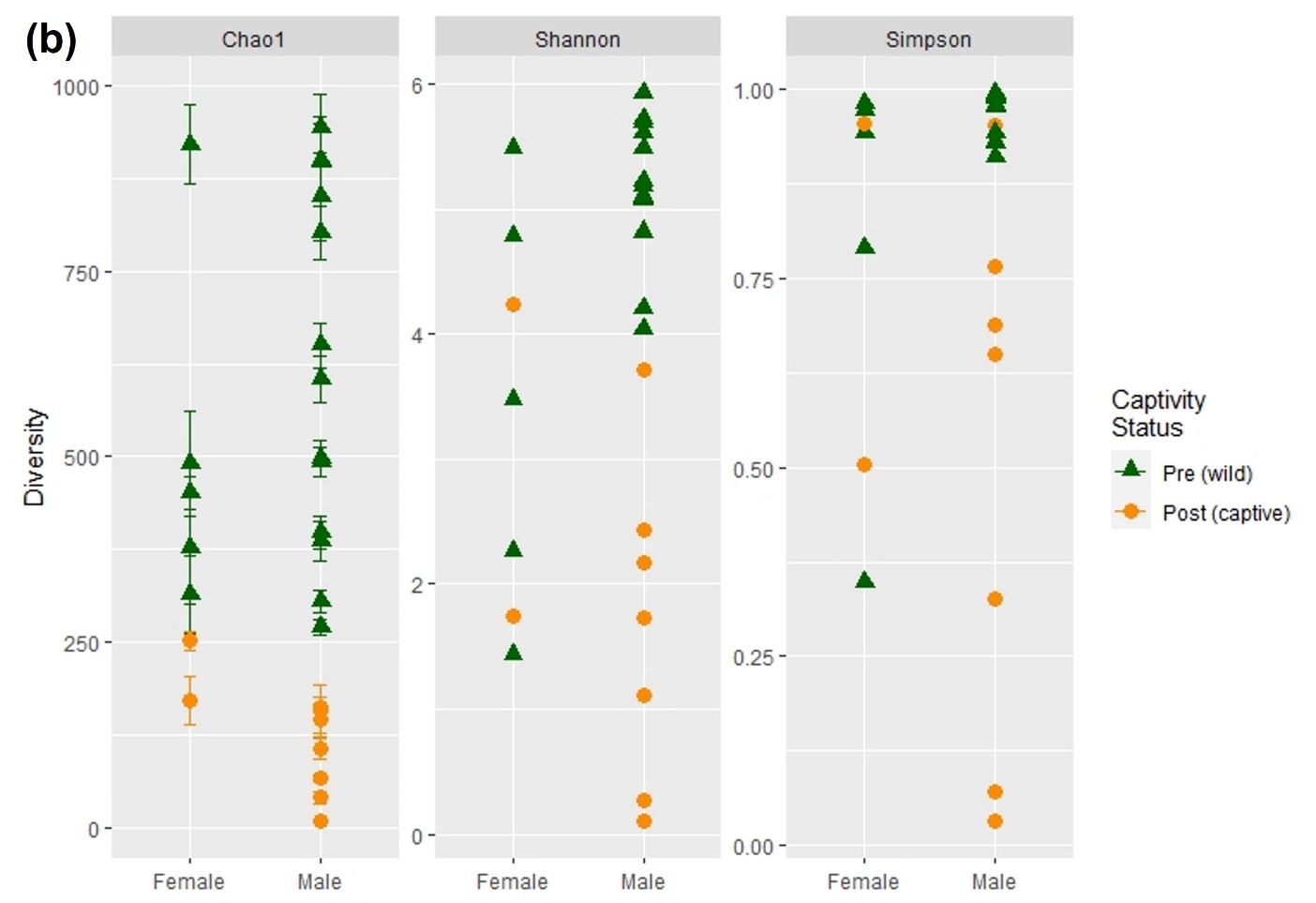


**Figure S1.** **Rarefied alpha diversity.** Alpha diversity (Chao 1, non-parametric Shannon, and inverse Simpson) of house sparrow bacteria from cloacal swabs after rarefying data. House sparrow cloacae were sampled before (green triangles) and after (orange circles) exposure to captivity. **(a)** Neophobia phenotype. None of the alpha diversity metrics significantly differed between neophobic and non-neophobic samples but pre- captivity samples had significantly higher alpha diversity than post-captivity samples (all p ≤ 0.04). Final samples sizes were as follows: pre-captivity = 18 (n = 6 neophobic, 12 non-neophobic) and post-captivity = 9 (n = 4 neophobic, 5 non-neophobic). **(b)** Sex. Wild male house sparrow cloaca samples had significantly higher bacteria alpha diversity for non-parametric Shannon (p = 0.005) and inverse Simpson metrics (p = 0.02). Final sample sizes were as follows: pre-captivity (wild) male = 13; post-captivity (captive) male = 7; pre-captivity (wild) female = 5; post-captivity (captive) female = 2.


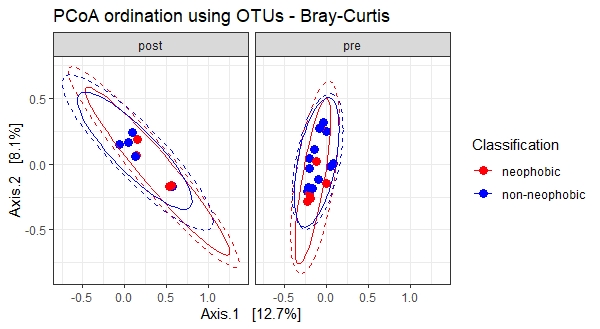


**Figure S2.** **Cloacal community composition** (beta diversity; non-rarefied data) of neophobic and non-neophobic house sparrows before (left frame) and after (right frame) captivity using non-rarefied data. Principal coordinates illustrate the shift between pre- (wild; right frame) and post-captivity (left frame) samples. We did not detect a difference between neophobic and non-neophobic phenotypes. Each point represents a cloacal sample from an individual. Increasing distance between points indicates increasing dissimilarity in cloacal community composition. The visualization using Jaccard distances is similar and thus not reported but can be produced using R code available in the supplementary material.


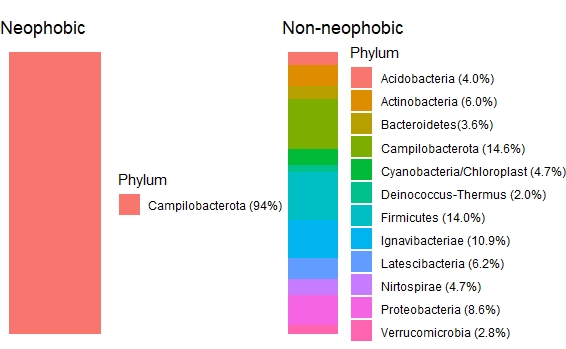


**Figure S3. Relative abundance of bacterial phyla** present in neophobic and non-neophobic house sparrow cloacae (non-rarefied). See supplementary Excel files for relative abundance between phenotypes for phyla, family, and genus upon rarefied and non-rarefied data.


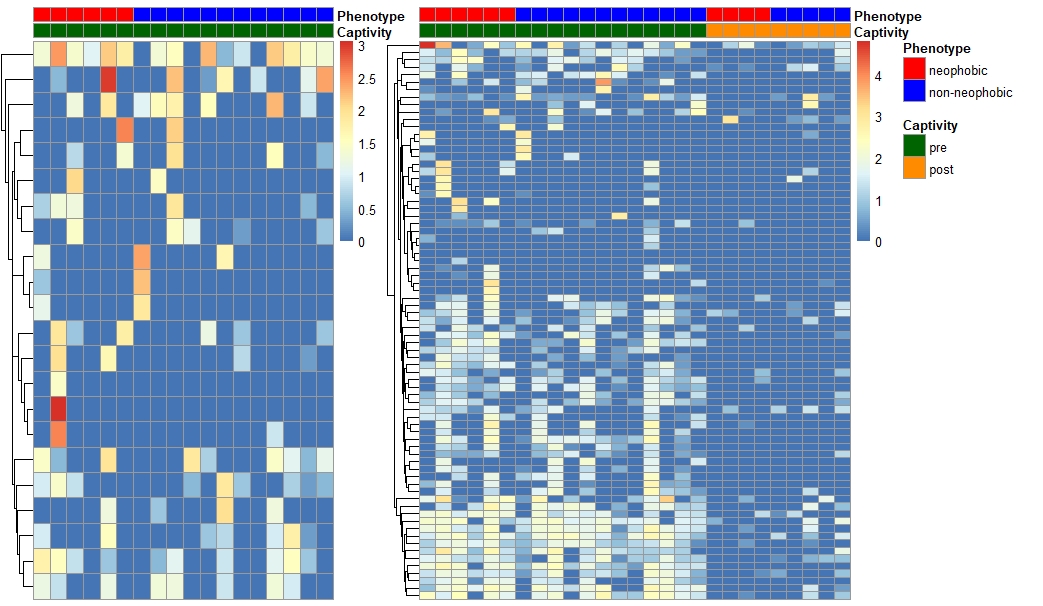


**Figure S4. Cloacal indicator OTUs (rarefied).** Heat map of the relative abundances of indicator OTUs from indicator species analysis associated with neophobic (left) and pre-captivity (right) house sparrow cloacae. There were no indicator OTUs associated with non-neophobic and captive house sparrow cloacae. Rows indicate unique OTUs and columns individual birds. The lowest taxonomic resolution that could be defined for OTU identification is listed in an Excel file (see Supplementary Material), as are indicator values, *p* values, FDR-corrected *p* values, positive predictive values, and sensitivity values.

**Supplementary Tables:**

**Table S1. Rarefied alpha diversity of house sparrow cloacal microbiomes was affected by captivity, but not neophobia phenotype.** **(a)** Results of linear mixed effects models for the effects of captivity and neophobia phenotype on cloacal alpha diversity. Results are reported for three different alpha diversity metrics: non-parametric Shannon, Chao, and inverse Simpson. Final samples sizes were as follows: pre-captivity = 18 (n = 6 neophobic, 12 non-neophobic) and post-captivity = 9 (n = 4 neophobic, 5 non-neophobic). Captivity effects are estimated for pre-captivity samples, for non-neophobic house sparrows and for females. **(b)** Results of linear models for the effect of sex on cloacal alpha diversity in wild (pre-captivity) samples (n = 13 males, 5 females). Effects are estimated for females, with respect to males. Statistically significant results are italicized.

|  | **Parameter estimate ± standard error** | **df** | **t** | **p** | **95% confidence interval** |
| --- | --- | --- | --- | --- | --- |
| **(a) overall *Non-parametric Shannon*** | | | | | |
| *captivity* | *3.5 ± 0.8* | *18.3* | *4.6* | *0.0002* | *2.0 – 4.9* |
| phenotype | 1.7 ± 0.8 | 22.0 | 2.1 | 0.05 | 0.1 – 3.4 |
| sex | -1.1 ± 0.6 | 16.4 | -2.0 | 0.06 | -2.2 – -0.1 |
| captivity × phenotype | -1.2 ± 1.0 | 16.9 | -1.2 | 0.3 | -3.0 – 0.8 |
| ***Chao*** | | | | | |
| *captivity* | *565.0* ± 143.6 | *22* | *3.9* | *0.0007* | *301.6 – 828.3* |
| phenotype | 80.0 ± 153.0 | 22 | 0.5 | 0.6 | -201.8 – 361.9 |
| sex | -55.4 ± 101.9 | 22 | -0.5 | 0.6 | -242.3 – 131.4 |
| captivity × phenotype | -160.2 ± 186.3 | 22 | -0.9 | 0.4 | -502.0 – 181.5 |
| ***Inverse Simpson*** | | | | | |
| *captivity* | *62.6* ± 27.4 | *13.8* | *2.3* | *0.04* | *8.0 – 113.0* |
| phenotype | 49.4 ± 31.2 | 21.3 | 1.6 | 0.1 | -22.7 – 114.6 |
| *sex* | *-64.5* ± 22.6 | *22.6* | *-2.9* | *0.01* | *-109.0 – -22.8* |
| captivity × phenotype | -3.1 ± 35.0 | 11.4 | -0.09 | 0.9 | -70.4 – 80.0 |
| **(b) wild sex effects *Non-parametric Shannon*** | | | | | |
| *sex* | *-1.7* ± 0.5 | *16* | *-3.3* | *0.005* | *-2.8 – -0.6* |
| ***Chao*** | | | | | |
| sex | -126.1 ± 133.3 | 16 | -0.9 | 0.4 | -408.7 – 156.4 |
| ***Inverse Simpson*** | | | | | |
| *sex* | *-72.1* ± 28.9 | *16* | *-2.5* | *0.02* | *-133.4 – -10.8* |

**Table S2.** **Resulting axis of principal coordinate analysis** (PCoA) using Bray-Curtis and Jaccard dissimilarity matrices upon rarefied and non-rarefied data, computed by phyloseq in R. **(a)** includes pre-captivity and post-captivity samples for ordination. **(b)** includes only pre-captivity samples.

| **(a) axis** (all samples) | **R^2^** | | | |
| --- | --- | --- | --- | --- |
|  | **Bray-Curtis** | | **Jaccard** | |
|  | **Not rarefied** | **Rarefied** | **Not rarefied** | **Rarefied** |
| 1 | 0.13 | 0.13 | 0.09 | 0.10 |
| 2 | 0.08 | 0.08 | 0.06 | 0.07 |
| 3 | 0.07 | 0.07 | 0.06 | 0.05 |
| **(b) axis** (pre-captivity) |  | | | |
| 1 | 0.13 | 0.14 | 0.10 | 0.10 |
| 2 | 0.11 | 0.09 | 0.09 | 0.07 |
| 3 | 0.08 | 0.08 | 0.07 | 0.07 |

**Table S3.** **Beta diversity (rarefied) of house sparrow cloacal microbiomes was affected by captivity, but not neophobia phenotype.** **(a)** Results of permutational multivariate analysis of variance (PERMANOVA) tests using Bray-Curtis and Jaccard dissimilarity that tested for effects of captivity, neophobia, and their interaction upon beta diversity. Each factor had a similar dispersion (Bray-Curtis all p > 0.17; Jaccard all p > 0.86). Final samples sizes were as follows: pre-captivity = 18 (n = 6 neophobic, 12 non-neophobic) and post-captivity = 9 (n = 4 neophobic, 5 non-neophobic). **(b)** Results of PERMANOVA tests using Bray-Curtis and Jaccard dissimilarity that tested for the effect of sex and phenotype upon beta diversity in wild (pre-captivity) samples (n = 13 males, 5 females). Abbreviations: SS = sum of squares; MSS = mean sum of squares; F = F statistic. Significant effects are italicized.

| **(a)** | **Bray-Curtis**  Figure 2a | | | | | **Jaccard** | | | | |
| --- | --- | --- | --- | --- | --- | --- | --- | --- | --- | --- |
|  | **df** | **SS** | **MSS** | **F** | **p** | **df** | **SS** | **MSS** | **F** | **p** |
| *captivity* | *1* | *1.0* | *1.0* | *2.6* | *0.001* | *1* | *0.8* | *0.8* | *1.9* | *0.001* |
| phenotype | 1 | 0.4 | 0.4 | 1.0 | 0.3 | 1 | 0.4 | 0.4 | 1.0 | 0.4 |
| sex | 1 | 0.5 | 0.5 | 1.2 | 0.1 | 1 | 0.5 | 0.5 | 1.1 | 0.2 |
| captivity × phenotype | 1 | 0.4 | 0.4 | 1.1 | 0.2 | 1 | 0.4 | 0.4 | 1.0 | 0.3 |
| residuals | 22 | 8.4 | 0.4 |  |  | 22 | 9.5 | 0.4 |  |  |
| total | 26 | 10.7 |  |  |  | 26 | 11.7 |  |  |  |
| **(b)** |  | | | | |  | | | | |
| *sex* | *1* | *0.5* | *0.5* | *1.5* | *0.01* | *1* | *0.5* | *0.5* | *1.3* | *0.008* |
| phenotype | 1 | 0.4 | 0.4 | 1.1 | 0.2 | 1 | 0.4 | 0.4 | 1.0 | 0.2 |
| residuals | 15 | 5.3 | 0.4 |  |  | 15 | 6.3 | 0.4 |  |  |
| total | 17 | 6.3 |  |  |  | 17 | 7.2 |  |  |  |
